# The Impact of Removal of Ovarian Hormones on Cholinergic Muscarinic Receptors: Examining Prepulse Inhibition and Receptor Binding

**DOI:** 10.3390/brainsci10020106

**Published:** 2020-02-17

**Authors:** Sarah S. Ch’ng, Adam J. Walker, Madeleine McCarthy, Thien-Kim Le, Natalie Thomas, Andrew Gibbons, Madhara Udawela, Snezana Kusljic, Brian Dean, Andrea Gogos

**Affiliations:** 1 Florey Institute of Neuroscience and Mental Health, University of Melbourne, Parkville, VIC 3010, Australia; 2 IMPACT, The Institute for Mental and Physical Health and Clinical Translation, School of Medicine, Deakin University and Barwon Health, Geelong, VIC 3220, Australia; 3 Affinity BIO, Scoresby, VIC 3179, Australia; 4 Department of Psychiatry, Monash University, Melbourne, VIC 3004, Australia; 5 Department of Nursing, University of Melbourne, Parkville, VIC 3010, Australia; 6 Centre for Mental Health, Swinburne University, Hawthorn, VIC 3122, Australia

**Keywords:** ovariectomy, female, rat, schizophrenia, PPI, CHRM1

## Abstract

Ovarian hormones, such as estrogens and progesterone, are known to exert beneficial effects on cognition and some psychiatric disorders. The basis of these effects is not fully understood, but may involve altered cholinergic neurotransmission. This study aimed to investigate how a lack of ovarian hormones would impact muscarinic receptor-induced deficits in prepulse inhibition (PPI) and muscarinic receptor density in several brain regions. Adult female rats were either ovariectomized, to remove the source of ovarian hormones, or left intact (sham-operated). PPI is a measure of sensorimotor gating that is typically impaired in schizophrenia patients, and similar deficits can be induced in rats by administering scopolamine, a muscarinic receptor antagonist. Our results revealed no significant effects of ovariectomy on PPI after saline or scopolamine treatment. Autoradiography was performed to measure cholinergic muscarinic receptor binding density using [^3^H]-pirenzepine, [^3^H]-AF-DX, and [^3^H]-4-DAMP, to label M_1_, M_2_/M_4_, and M_3_ receptors, respectively. We examined the amygdala, caudate putamen, dorsal hippocampus, motor cortex, retrosplenial cortex, and ventromedial hypothalamus. There were no significant group differences in any region for any muscarinic receptor type. These results suggest that removing peripheral ovarian hormones does not influence the cholinergic muscarinic receptor system in the context of PPI or receptor binding density.

## 1. Introduction

Current data suggest that female ovarian hormones can improve cognitive function [1,2,3] and reduce symptom severity in neuropsychiatric disorders, such as schizophrenia [4]. For instance, epidemiological evidence indicates that, compared to women, there is a higher incidence and earlier onset of schizophrenia in men [5]; the latter phenomenon is consistent across cultures and independent of the diagnostic classification system used [6,7,8]. There is also evidence to suggest that the impact of ovarian hormones on the symptoms of schizophrenia varies across the reproductive lifespan of women, which is underscored by changing levels of hormones, including the main ovarian hormones, estrogen and progesterone [8,9,10]. Overall, these factors lead to a less severe course of illness, milder symptoms, and superior treatment outcomes in women. 

Cholinergic signaling plays a crucial role in multiple cognitive processes, including learning, memory, and sensory perception [11]. Converging lines of evidence have implicated muscarinic cholinergic signaling in the pathophysiology of schizophrenia. In patients with schizophrenia, in vivo muscarinic receptor availability was reduced [12,13], and postmortem radioligand binding studies using [^3^H]-pirenzepine, which label muscarinic M_1_ and M_4_ receptors, have indicated that the densities of these receptors were decreased in the prefrontal cortex, anterior cingulate cortex, hippocampus, and caudate putamen of schizophrenia patients [14,15,16,17,18]. Notably, decreased levels of both M_1_ mRNA levels [17,19,20] and M_1_ receptor protein, but not M_2_, M_3_, or M_4_ receptor protein [17,21], were observed in the dorsolateral prefrontal cortex and superior prefrontal gyrus from people with schizophrenia. These data suggest that low levels of cortical M_1_ receptors are a significant component of the pathophysiology of schizophrenia. 

Scopolamine is a nonselective muscarinic receptor antagonist that induces a host of cognitive and psychotic symptoms referred to as “anti-muscarinic syndrome”, which resembles some clinical aspects of schizophrenia [22,23]. In rodents, administration of scopolamine is known to induce deficits in prepulse inhibition (PPI) [24,25,26], which has been proposed as an antimuscarinic model of schizophrenia [27]. PPI is the attenuation of the acoustic startle response to an intense audiogenic stimulus (pulse) by a preceding, weaker acoustic stimulus (prepulse). PPI is used as an operational measure of sensorimotor gating, a process which is deficient in schizophrenia, and can be measured with analogous methods in rodents [28,29,30]. 

We and others have previously investigated the effects of ovariectomy, ovarian hormones, and their synthetic analogues in PPI [31,32,33,34,35]. For example, while ovariectomy had no effect on PPI [31,32,33], treatment with the estrogen, 17β-estradiol, could reverse PPI deficits that were induced by serotonin 1A (5-HT_1A_) receptor agonists in both female rats [32] and healthy women [36]. Specifically, chronic high-dose estradiol treatment prevented the disruptions in PPI induced by the 5-HT_1A_ receptor agonist 8-hydroxy-dipropylaminotertralin (8-OH-DPAT) in rats [32]. Similarly, estradiol treatment in healthy women abolished the deficits in PPI induced by the partial 5-HT_1A_ receptor agonist, buspirone [36]. Subsequent pharmacological studies in rats indicated that chronic estradiol treatment inhibited the effects of 8-OH-DPAT and the dopamine D_1_/D_2_ receptor agonist, apomorphine, on PPI, and that the protective effects of estrogen on PPI were at least partly dependent on dopamine D_2_ receptor signaling down-stream of 5-HT_1A_ receptor signaling [37]. Collectively, these findings implicate ovarian hormones, particularly estradiol, in the regulation of PPI. The impact of removal of ovarian hormones on PPI deficits induced by cholinergic muscarinic receptor agonists has not been previously examined.

Given that both muscarinic receptors and ovarian hormones are implicated in the mediation of PPI, the aim of this study was to assess the effects of ovariectomy on scopolamine-induced deficits in PPI and binding density of muscarinic receptors in the CNS in rats. 

## 2. Materials and Methods

### 2.1. Animals

In total, 35 female Sprague-Dawley rats were used in this study. Rats were obtained from a commercial supplier (Animal Resources Centre; Perth, WA, Australia) when they were 9–10 weeks old and housed in open top cages in groups of 2–3 per cage with access to standard rat chow and water ad libitum. Rats were housed on a 12-hour light-dark cycle (lights on at 6 a.m.) at constant temperature of 23 ± 2 °C. All experimental procedures were approved by the Florey Institute of Neuroscience and Mental Health Animal Ethics Committee and were performed in accordance with the Australian Code of Practice for the Care and Use of Animals for Scientific Purposes.

### 2.2. Ovariectomy

At 11-12 weeks of age, rats were either left intact (sham-operated) or ovariectomized (OVX; *n* = 10 per group) as reported previously [37]. For surgical procedures, rats were anaesthetized with isoflurane/oxygen gas mixture and injected with analgesic anti-inflammatory agent, carprofen (Rimadyl, Pfizer, 5 mg/kg; West Ryde, NSW, Australia), to reduce postoperative pain. A 2–3 cm incision was made through the skin and abdominal wall on the lower medial region of the dorsal surface. The ovaries were located, and the ovaries were then bilaterally removed in the OVX rat group only. The wound was closed using two surgical sutures and covered with antiseptic cream (Betadine, povidone-iodine 10%, Faulding Consumer; Salisbury, SA, Australia). Rats were given 10 days to recover before further experimentation.

### 2.3. Prepulse Inhibition (PPI)

PPI was measured using automated startle chambers (San Diego Instruments, San Diego, CA, USA), as described previously [37]. In brief, rats were placed in a transparent Plexiglas cylinder that sat within a sound-attenuating cabinet. Audiogenic stimuli were delivered with a speaker, and whole-body startle responses were measured using the automated SR-Lab software (San Diego Instruments). The 30-minute session consisted of 80 trials presented at variable intervals (8–27 s), 8 no tone (i.e., only background, 70 dB), 32 pulse-alone (4 blocks of 8 × 115 dB) trials, and 40 prepulse trials (8 of each: 2, 4, 8, 12, or 16 dB above background followed 100 ms later by the 115 dB pulse). Startle data were measured using all four blocks of pulse-alone trials. The percentage of PPI was calculated as the difference in amplitude between the startle response to the pulse-alone trials and the prepulse-pulse trials, divided by the response to the pulse-alone trial × 100%.

Saline or 0.3 mg/kg scopolamine (Scopolamine hydrobromide trihydrate; Sigma-Aldrich, St. Louis, MO, USA) was administered subcutaneously 30 min prior to PPI testing. Scopolamine was dissolved in saline to a dose based on previous literature [25,38] and administered in a volume of 1 ml/kg. Using a randomized, crossover protocol, rats received both treatments with 3–4 days allowed between each experiment. 

### 2.4. Autoradiography

**Figure 1:**
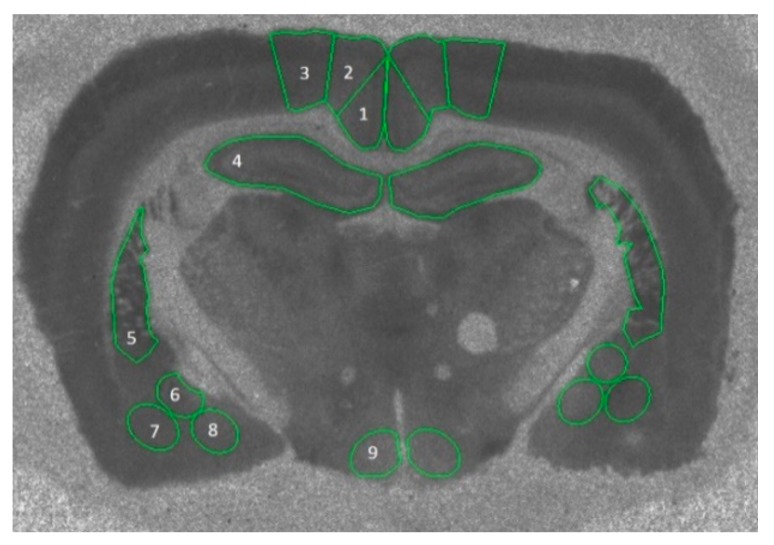
Example autoradiographic image of [^3^H]-AF-DX binding showing the traced outlines of the nine regions of interest: (1) retrosplenial cortex (granular), (2) retrosplenial cortex (dysgranular), (3) motor cortex (M1, M2), (4) dorsal hippocampus, (5) caudate putamen, (6) central amygdala, (7) basolateral amygdala, (8) medial amygdala, and (9) ventromedial hypothalamus.

In a separate cohort of intact (sham-operated) (*n* = 7) or OVX (*n* = 8) rats (procedure as described above), frozen whole brains were blocked, and serial coronal 20 μm sections were sectioned on a cryostat (Leica CM18-50, Leica Microsystems Nussloch GmbH, Germany) and thaw mounted onto gelatinized microscope slides. Sections were collected aiming for bregma −2.76 mm [39], such that the following regions could be traced for the left and right hemispheres: retrosplenial cortex (granular and dysgranular), motor cortex (M1 and M2 combined), dorsal hippocampus, ventral caudate putamen, central amygdala, basolateral amygdala, medial amygdala, and ventromedial hypothalamus (Figure 1). Due to issues with sectioning and damaged tissue, we were unable to collect a measurement for each region for each rat, thus the final sample was *n* = 3–8 per group. 

**Figure 2:**
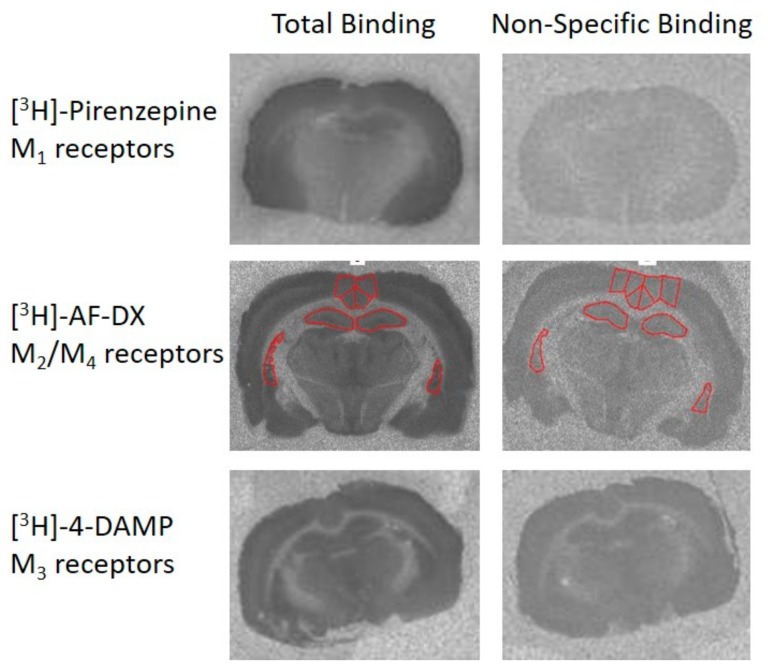
Representative autoradiographic images showing total binding (left panels) and nonspecific binding (right panels). Three tritiated radioligands were used with the protocols described in Section 2.4.

Under the conditions used in this study, [^3^H]-pirenzepine would provide a measure of M_1_ receptors [40], [^3^H]-AF-DX 384 would provide a measure of M_2_ and M_4_ receptors [41], and [^3^H]-4-DAMP (4-diphenylacetoxy-N-methylpiperidine methiodide) would provide a measure of M_3_ receptors [42]. For all three radioligands (PerkinElmer, Waltham, MA, USA), three sections per animal were used to measure total radioligand binding, and two sections per animal were used to measure nonspecific binding (Figure 2).

For [^3^H]-pirenzepine binding, tissue sections were incubated in assay buffer (10 mM KH_2_PO_4_, 10 mM Na_2_HPO_4_; pH 7.4) containing 25 nM [^3^H]-pirenzepine in the presence or absence of 1 μM QNX (3-quinuclidinyl xanthene-9-carboxylate hemioxalate; Sigma-Aldrich) for 30 min at room temperature. [^3^H]-AF-DX 384 binding was measured after incubating tissue sections in assay buffer (10 mM KH_2_PO_4_, 10 mM Na_2_HPO_4_; pH 7.4) for 30 min at room temperature. The tissue sections were then dipped in distilled water and air-dried. Sections were next incubated in assay buffer containing 24 nM [^3^H]-AF-DX 384 in the absence (total binding) or presence (nonspecific binding) of 1 μM tropicamide (Tocris Bioscience, Bristol, UK) for 60 min at room temperature. For both radioligands, tissue sections were then washed twice for 2 min in ice-cold assay buffer, dipped in ice-cold distilled water, dried, partially fixed in paraformaldehyde vapor overnight, and then apposed to a BAS-TR2025 plate (Fujifilm, Tokyo, Japan) with autoradiographic tritium standards (American Radiolabeled Chemicals, St. Louis, MO, USA) for 3 ([^3^H]-pirenzepine) or 7 ([^3^H]-AF-DX 384) days.

For [^3^H]-4-DAMP binding, tissue sections were incubated in assay buffer (50 mM Tris-HCl; pH 7.4) for 15 min at room temperature, dipped in distilled water, and air-dried. Sections were then incubated in assay buffer containing 60 nM [^3^H]-4-DAMP and 1 μM pirenzepine dihydrochloride (to displace M_1_ receptors) in the absence (total binding) or presence (nonspecific binding) of 10 μM 4-DAMP mustard for 60 min at room temperature. After radioligand binding, slides were washed twice for 5 min in ice-cold assay buffer, dipped in ice-cold distilled water, dried, partially fixed overnight in paraformaldehyde vapor, and apposed to a BAS-TR2025 plate with autoradiographic tritium standards for 3 days.

In all cases, the BAS-TR2025 plates were scanned in a BAS 5000 high resolution phosphoimager (Fujifilm, Tokyo, Japan). The resulting images were analyzed using AIS imaging software (Imaging Research, St. Catharines, ON, Canada). Radioligand binding was measured as an integrated measurement of signal intensity across the entire region of binding, including both hemispheres. Signal intensities were calibrated against the standards and expressed as the average amount of total bound radioligand (fmol/mg) estimated tissue equivalent (ETE) minus the average nonspecific binding for each rat [43].

### 2.5. Statistical Analysis

All data are expressed as mean ± standard error of the mean (S.E.M.) and were analyzed using IBM SPSS Statistics for Windows, Version 26 (IBM Corp., Armonk, NY, USA). Differences between groups were considered significant if *p* < 0.05. Group differences in body and uterus weights were analyzed using unpaired *t*-tests. For PPI, a 2 group (intact, OVX) x 2 drug (saline, scopolamine) × 5 prepulse intensity (PP2–PP16) three-way repeated-measures analysis of variance (ANOVA) was used. For startle amplitude, a 2 group (intact, OVX) × 2 drug (saline, scopolamine) × 4 block (4 × 8 pulse-alone trials) three-way ANOVA was used. For graphical presentation only, average startle amplitudes were used. Group differences in muscarinic receptor binding density were analyzed using separate unpaired *t*-tests for each receptor in each brain region.

## 3. Results

### 3.1. Ovariectomy

At the time of surgery, body weights did not differ between the groups (Intact: 258 ± 6 g, OVX: 254 ± 4 g; *t*(18) = 0.47, *p* = 0.64). Consistent with our prior studies [37], at the end of experimentation, OVX rats had significantly greater body weight (Intact: 307 ± 11 g, OVX: 361 ± 13 g; *t*(18) = −3.22, *p* = 0.005) than intact rats. OVX rats also had significantly smaller uteri when expressed as an absolute weight (Intact: 0.61 ± 0.04 g, OVX: 0.11 ± 0.00 g; *t*(18) = 11.30, *p* < 0.001) or as a percentage of body weight (Intact: 0.20 ± 0.01 %, OVX: 0.03 ± 0.00 %; *t*(18) = 11.33, *p* < 0.001). These findings confirmed the effectiveness of the OVX surgery.

### 3.2. Prepulse Inhibition

**Figure 3:**
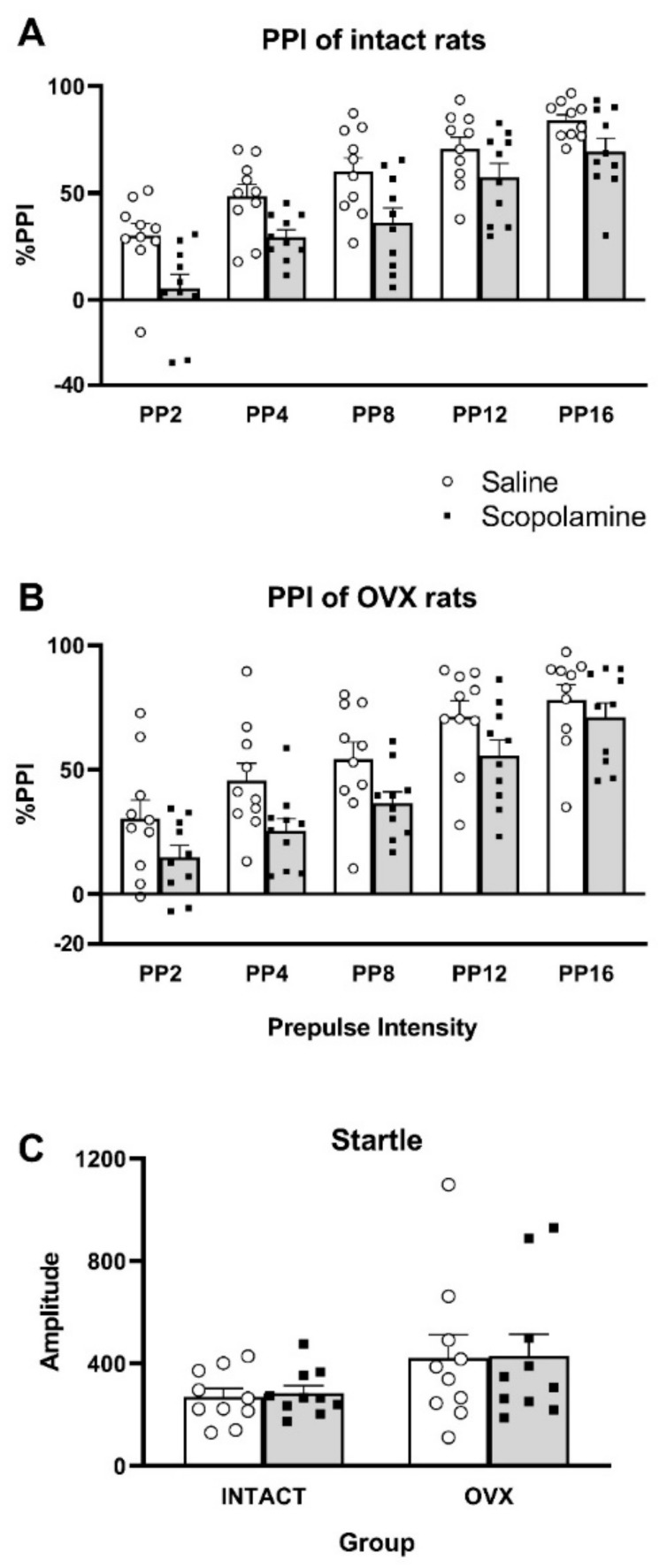
Prepulse inhibition (PPI; graphs **A** and **B**) and startle responses (graph **C**) in intact or ovariectomized (OVX) rats (*n* = 10 per group). Rats were tested after treatment with saline (white bars) or 0.3 mg/kg scopolamine (grey bars). Individual data are depicted by open circles (saline) or black squares (scopolamine). %PPI refers to the difference in startle between the pulse-alone trials and the prepulse-pulse trials, divided by the response to the pulse-alone trial × 100%. Prepulse intensity (PP) refers to the trials that were 2, 4, 8, 12, or 16 dB above the 70 dB background noise followed 100 ms later by the 115 dB startling pulse. Startle amplitude refers to the average of the four blocks of eight 115 dB startling pulses. Bars represent mean ± S.E.M.

When comparing PPI in intact and OVX rats that were administered saline or 0.3 mg/kg scopolamine (2 group × 2 dose × 5 prepulse intensity ANOVA; Figure 3), the main effect of prepulse intensity was observed (*F*(4,72) = 94.18, *p* < 0.001), reflecting an increase in PPI with increasing prepulse intensity. The main effect of drug was observed (*F*(1,18) = 51.20, *p* < 0.001), reflecting the disruption of PPI caused by scopolamine treatment in intact rats (*F*(1,9) = 45.41, *p* < 0.001) and OVX rats (*F*(1,9) = 15.49, *p* = 0.003; Figure 3). No main effect of group, or any interactions of group and drug or prepulse intensity, were detected, reflecting a similar disruption in PPI in both groups.

When comparing startle amplitude between groups (2 group × 2 drug × 4 block ANOVA), no significant effects or interactions were observed, reflecting similar startle amplitudes in both groups, with or without scopolamine and over the duration of the PPI session (Figure 3). Thus, the PPI results were not affected by a change in startle amplitude.

### 3.3. Receptor Binding Autoradiography

There were no significant differences in [^3^H]-pirenzepine, [^3^H]-AF-DX, or [^3^H]-4-DAMP binding density in any of the nine brain regions between intact and OVX rats (Table 1). A trend for reduced [^3^H]-AF-DX binding density was observed in the hippocampus of OVX rats compared to intact rats (*t*(6) = 2.30, *p* = 0.061). There was also a trend for reduced [^3^H]-4-DAMP binding density in the central amygdala of OVX rats compared to intact rats (*t*(7) = 2.19, *p* = 0.065). 

## 4. Discussion

In this study, we aimed to investigate the effects of ovariectomy on scopolamine-induced PPI deficits and cholinergic muscarinic receptor binding density to assess potential interactions between ovarian hormones and muscarinic receptors. We did not observe any effects of ovariectomy on scopolamine-induced impairments in PPI, nor did we detect any significant changes in the binding density of M_1_, M_2_/M_4_, and M_3_ receptors in the amygdala, caudate putamen, dorsal hippocampus, motor cortex, retrosplenial cortex, or ventromedial hypothalamus of OVX rats relative to intact rats. These results suggest that muscarinic receptor antagonist-induced PPI deficits are independent of ovarian hormone status. To our knowledge, this study is the first to examine scopolamine-induced PPI deficits in female rats and to systematically assess the effects of eliminating circulating ovarian hormones on M_1_, M_2_/M_4_, and M_3_ receptor density in the female rat brain. 

Past research has highlighted an interaction between estrogen and the muscarinic system. For instance, ovariectomy attenuates high-affinity choline uptake and acetylcholine synthesis in cerebral cortical synaptosomes [44]. Estrogen receptors are densely expressed in the basal forebrain, which provides major cholinergic input to the hypothalamus, hippocampus, and cortex [45,46]. Further, muscarinic receptor binding fluctuates across the estrous cycle [47], and ovariectomy upregulates M_4_ receptors in the hippocampus, frontal cortex, and hypothalamus, an effect that was normalized by estrogen, but not progesterone, treatment [48]. In humans, chronic estrogen therapy in women was associated with greater cholinergic responsivity and significantly higher M_1_ and M_4_ receptor density compared to that in women who were naïve to estrogen therapy [49,50]. Based on these findings, we hypothesized that removal of estrogen would modulate muscarinic receptor expression in nine specific brain regions, namely, the granular and dysgranular retrosplenial cortex; motor cortex; dorsal hippocampus; caudate putamen; amygdaloid regions including the central, basolateral, and medial nuclei; and ventromedial hypothalamus. These regions were selected for anatomical and functional reasons, as they express a high density of muscarinic receptors and are implicated in various sensorimotor and cognitive processes. For instance, the retrosplenial cortex plays a crucial role in spatial and episodic memory, navigation, and planning for the future [51]. The retrosplenial cortex receives dense cholinergic innervation from the basal forebrain, and the loss of such cholinergic inputs induces upregulation of muscarinic receptors in this region [52]. In the dorsal hippocampus, more than 90% of nonpyramidal/GABAergic neurons express muscarinic receptors [53]. Moreover, muscarinic receptor signaling in the dorsal hippocampus is critical for memory consolidation [54]. Indeed, muscarinic receptor levels are decreased in the hippocampal formation of schizophrenia patients relative to healthy controls [15], most likely due to low levels of M_4_ receptor expression [55], whilst [^3^H]-AF-DX binding is reduced in the caudate putamen of schizophrenia patients [41] in the absence of changes in M_1_ and M_2_ receptor expression [20]. The amygdaloid complex, especially the central amygdala, also expresses a high density of muscarinic receptors [56]. Estradiol treatment dose-dependently increased muscarinic receptor binding in the ventromedial hypothalamus of OVX female rats, whereas muscarinic receptor binding was not altered in vehicle-treated OVX female rats or estradiol-treated male rats [57]. Although the above literature highlights an interaction between estrogen and the muscarinic system, the precise role of estrogen and other ovarian hormones in cholinergic signaling is unclear and likely involves a complex modulatory role of ovarian hormones on multiple systems.

In the present study, we did not observe any significant changes in M_1_, M_2_/M_4_, or M_3_ receptor binding density in OVX rats relative to intact rats in any of the brain regions examined, suggesting that removal of ovarian hormones does not directly impact muscarinic receptor density in these brain regions in adult female rats. Studies have assessed the effects of ovariectomy on the expression of muscarinic receptor subtypes (M_1_ to M_5_) but have largely focused on the rat hippocampus [58,59,60]. In contrast to our findings, one study reported that ovariectomy augmented the protein expression of all muscarinic receptor subtypes compared to control in whole hippocampal homogenates from Wistar rats [58]. Various methodological differences may explain this discrepancy; our study measured individual muscarinic receptors, in the dorsal hippocampus, using tissue sections from Sprague-Dawley rats. Nevertheless, we observed a trend for reduced M_2_/M_4_ receptor binding density in the hippocampus of OVX rats relative to intact rats. Pharmacological and behavioral studies have implicated the importance of M_2_ receptors in learning and memory task performance [61,62]. The trend for reduced hippocampal M_2_/M_4_ receptor binding partly supports past observations that ovariectomy affects hippocampal-dependent learning and memory in rats. Indeed, the positive effects of estrogen on working memory are dependent on M_2_ receptor hippocampal signaling [63]. 

In humans, ovarian hormones have been demonstrated to affect PPI [36], with lower PPI in females than in males, and reduced PPI in women during the luteal phase when circulating estradiol and progesterone are elevated [64,65,66]. We have previously demonstrated that estradiol treatment has a protective effect on disruptions of PPI induced by the 5-HT_1A_ receptor agonist; 8-OH-DPAT; and dopamine receptor agonist, apomorphine [32,36]. We therefore assessed whether removal of the major source of circulating estrogen would impact deficits in PPI induced by the nonselective muscarinic receptor antagonist, scopolamine. However, we did not observe any differences in scopolamine-induced deficits in PPI between OVX and intact rats. Our results suggest that scopolamine-induced impairments in PPI are not mediated by ovarian hormones, given that the elimination of circulating ovarian hormones did not significantly alter PPI. The absence of behavioral effects was consistent with the lack of alterations in muscarinic receptor binding density observed in this study. Ovariectomy removes the main source of estradiol, progesterone, and other ovarian hormones from the periphery. Thus, the lack of effect observed here could be due to the role of progesterone (or other hormones) masking the effects of estradiol, as progesterone could be exerting contrasting effects to those induced by estradiol [10]. Additionally, central production of these hormones may have partly compensated for the reduction in peripheral levels of these hormones. Further research is needed to determine the exact role of each ovarian hormone. It would also be of interest to determine if ovarian hormones influence muscarinic receptor activation, as the M_1_/M_4_ receptor agonist, xanomeline, has been shown to be efficacious in the treatment of schizophrenia [67].

A limitation of the binding results is that several tissue samples were damaged, and we were unable to collect a measurement for every single region in each rat. As such, group sample sizes were unequal and some groups had small sample size (*n* = 3–8), which limits the generalizability of these findings. Future research should repeat these experiments with larger sample sizes to increase statistical power and improve signal detection. Another limitation is that we did not control for estrous cycle stage in the control rats.

## 5. Conclusions 

In conclusion, this study demonstrated that ovariectomy does not influence the cholinergic muscarinic receptor system in the context of PPI or receptor binding density. Our results suggest that removing peripheral ovarian hormones do not affect scopolamine-induced disruptions in PPI or muscarinic receptor binding density. Further, it is possible that ovarian hormones may have effects on cognition through pathways other than the cholinergic muscarinic receptor system. 

## Figures and Tables

**Table 1:** Mean (S.E.M.) cholinergic muscarinic receptor binding density (fmol/mg estimated tissue equivalent) in intact and ovariectomized (OVX) rats in nine brain regions (*n* = 3–8 per group). [^3^H]-Pirenzepine, [^3^H]-AF-DX 384, and [^3^H]-4-DAMP were used to label M_1_, M_2_/M_4_, and M_3_ receptors, respectively.

	[^3^H]-Pirenzepine		[^3^H]-AF-DX		[^3^H]-4-DAMP	
	INTACT	OVX	INTACT	OVX	INTACT	OVX
**Retrosplenial cortex (granular)**	66.4 (1.7)	64.9 (2.3)	77.1 (3.0)	75.1 (3.4)	71.4 (3.6)	79.0 (4.9)
**Retrosplenial cortex (dysgranular)**	89.7 (3.4)	84.2 (6.3)	81.0 (4.3)	82.3 (4.8)	91.8 (4.6)	90.5 (6.4)
**Motor cortex (M1, M2)**	105.5 (5.2)	108.1 (5.8)	92.6 (6.5)	92.2 (3.0)	124.0 (13.4)	134.8 (17.8)
**Dorsal hippocampus**	93.3 (14.4)	82.2 (11.4)	71.9 (2.6)	61.2 (4.4)	82.2 (5.8)	76.6 (3.3)
**Caudate putamen (ventral)**	91.4 (9.0)	94.4 (7.5)	89.6 (2.8)	96.9 (4.1)	120.2 (16.7)	122.7 (16.9)
**Central amygdala**	97.5 (9.7)	82.3 (2.2)	68.2 (2.6)	60.7 (4.4)	78.1 (2.4)	65.8 (4.6)
**Basolateral amygdala**	126.3 (7.5)	114.2 (2.5)	96.7 (7.4)	90.5 (6.6)	77.8 (5.1)	81.6 (6.6)
**Medial amygdala**	73.0 (3.1)	70.3 (3.1)	58.1 (2.4)	54.5 (3.1)	72.4 (5.2)	61.8 (4.7)
**Ventromedial hypothalamus**	18.9 (1.3)	19.7 (2.6)	31.3 (3.3)	27.5 (5.4)	-	-
